# Acute Effect of Intranasal Insulin on Food Intake Among Middle-Aged African American Adults: The FIINAAL Study

**DOI:** 10.1210/jendso/bvaf138

**Published:** 2025-08-25

**Authors:** Mia M Goodson, Kathryn L Gwizdala, Isabella R Manrique, Arushi Rao, Robbie Beyl, Corby K Martin, Simon Firmin, Vanessa Salceanu, Robert L Newton, Owen T Carmichael

**Affiliations:** Department of Biomedical Imaging, Pennington Biomedical Research Center, 6400 Perkins Road, Baton Rouge, LA 70808, USA; Department of Biomedical Imaging, Pennington Biomedical Research Center, 6400 Perkins Road, Baton Rouge, LA 70808, USA; Department of Biomedical Imaging, Pennington Biomedical Research Center, 6400 Perkins Road, Baton Rouge, LA 70808, USA; Department of Biomedical Imaging, Pennington Biomedical Research Center, 6400 Perkins Road, Baton Rouge, LA 70808, USA; Department of Biostatistics, Pennington Biomedical Research Center, 6400 Perkins Road, Baton Rouge, LA 70808, USA; Department of Clinical Science, Pennington Biomedical Research Center, 6400 Perkins Road, Baton Rouge, LA 70808, USA; Department of Biomedical Imaging, Pennington Biomedical Research Center, 6400 Perkins Road, Baton Rouge, LA 70808, USA; Department of Biomedical Imaging, Pennington Biomedical Research Center, 6400 Perkins Road, Baton Rouge, LA 70808, USA; Department of Population and Public Health, Pennington Biomedical Research Center, 6400 Perkins Road, Baton Rouge, LA 70808, USA; Department of Biomedical Imaging, Pennington Biomedical Research Center, 6400 Perkins Road, Baton Rouge, LA 70808, USA

**Keywords:** intranasal insulin, Alzheimer disease, health disparities

## Abstract

**Context:**

Intranasal insulin has emerged as a promising potential treatment for cognitive decline. However, African American adults are under-represented in this research area, and unintentional weight loss is a possible detrimental side effect.

**Objective:**

We assessed effects of acute intranasal insulin exposure on food intake and appetite-related constructs among middle-aged, obese, cognitively normal African American adults. We hypothesized that intranasal insulin would result in fewer calories consumed, greater feelings of fullness, and less hunger compared to placebo.

**Method:**

A total of 39 participants received intranasal doses of Novolin-R (40 IU) and a saline placebo on separate days in a double-blind, counterbalanced, crossover design, with 3-day, eucaloric, nutritionally balanced diets preceding each dose. Doses were preceded by a 4-hour fast and followed by a test lunch. Visual analog scales (VAS) were used to assess appetite immediately before and after each dose, and after each lunch. Mixed effects linear model *t* tests were used to compare questionnaires and lunch intake between insulin and placebo.

**Results:**

There were no significant differences in food intake between conditions. However, feelings of fullness were significantly greater immediately after insulin compared to placebo. In addition, the desire to consume sweet foods decreased significantly more after insulin than after placebo.

**Conclusion:**

Acute intranasal insulin was associated with a reduced desire for sweet foods and with increased feelings of fullness, but not reduced food intake, among middle-aged African American adults. Eating behavior and appetite changes should be explored further as possible side effects of intranasal insulin treatment for cognitive decline in diverse populations.

More than 5 million Americans are currently living with Alzheimer disease (AD) [1]. It is the sixth leading cause of mortality in the United States, resulting in more than 80 000 deaths per year and treatment costs of over 200 billion dollars per year [1]. One proposed therapeutic approach to AD is direct delivery of insulin to the brain, following multiple lines of study that suggest that dysregulation of brain insulin production, delivery, receptor binding, signaling, and/or downstream events may be dysregulated along the pathway to AD [2-11]. Insulin delivered intranasally has been shown to gain direct brain access [6], with minimal effects on circulating insulin levels. It has also been shown to enhance attention and everyday functioning in older adults along a spectrum from cognitive health to dementia [5], and some studies suggest that intranasal insulin may modulate plasma beta amyloid and other AD biomarkers [12, 13]. Because intranasal insulin has shown promise as a therapeutic agent across a range of individuals from preclinical AD to AD dementia, interest in its further development has been strong.

One key limitation of the current literature on intranasal insulin as an AD therapeutic is a lack of clarity about unintentional weight loss as a side effect. Prior studies have been mixed, with several suggesting that a single dose is associated with reductions in food intake [14-16], and others reporting no such effect [17]. Studies of the acute effects of intranasal insulin on food cue processing have similarly been mixed [18-21]. In addition, studies of intranasal insulin effects on metabolic rate, reward processing generally, and processing of rewarding stimuli outside of food have been relatively scarce but support significant effects [22-25]. The small number of studies to date featuring chronic dosing have also been mixed regarding weight loss [26, 27]. Basic science studies have also suggested that insulin in the brain inhibits anabolic signaling pathways that encourage weight gain, while activating catabolic neuronal systems that cause either anorexia or increased energy expenditure [28]. However, to our knowledge, weight loss and its drivers (including reduced food intake) have not been explored as potential side effects of intranasal insulin therapy in older adults, especially among those who are under-represented in AD research.

Another limitation of the intranasal insulin literature is a relative lack of data from middle-aged individuals (for example, those aged 50 to 60) who may be at the earliest stages of measurable cognitive decline [29, 30] and AD-related neurobiological processes [31]. Individuals in this age range have been hypothesized to be the most likely to benefit from AD therapies because neurobiological injury processes associated with AD have been active for a shorter period of time, and thus the amount of intact brain tissue is larger than that of corresponding older adults. Yet to date, the typical intranasal insulin study has focused on older adults; for example, cohorts with a mean age in the 70s [32].

Finally, while existing data supports the concept of intranasal insulin as an AD therapeutic, the sheer number of individuals identifying as African American in these studies is small, typically constituting 5% of study populations or less [33]. The lack of representation for African Americans is caused by a constellation of barriers, including strict inclusion criteria, participant burden [34], lack of trust in the medical field [35-37], fear of harms and exploitation, low health literacy, randomization concerns, and a feeling that there is no personal benefit to participation [38]. These factors have likely contributed to distrust of researchers, reduced interest in participation, and subsequent lack of familiarity with the clinical research process. However, individuals who identify with racial and ethnic minority groups express willingness to participate in research studies [35, 37, 39, 40], and certain characteristics, such as being socially active and having one's own transportation [40], are associated with greater participation. This suggests that remediating the under-representation of African Americans in AD research is an attainable goal.

The purpose of this study was to assess the effects of acute intranasal insulin exposure on food intake and self-reported appetite, which could be drivers of weight loss, among middle-aged, cognitively normal African American individuals. Our double-blind, placebo-controlled, crossover-design study compared a single acute 40 IU dose of intranasal Novolin-R to saline placebo in terms of effects on ingestive behavior questionnaires and food intake at a post-dose ad libitum buffet lunch. We hypothesized that acute intranasal insulin would result in the consumption of fewer calories, greater feelings of satiety and fullness, and less hunger compared to placebo.

## Methods

### Recruitment Methods

Participants completed web and/or phone screening to assess eligibility, followed by an in-person screening visit. Individuals were eligible if they identified as African American, were between 45 and 65 years of age, were willing to provide written informed consent, and were able to read and speak English. Participants were excluded for the following reasons: clinical history of type 1 or 2 diabetes, self-reported pregnancy or attempting to become pregnant, history of sensitivity to glutaraldehyde (an antimicrobial agent used to sterilize the intranasal insulin inhalation device), current prescription of any medication that could interact with intranasal insulin, clinical history of respiratory or nasal illness that could interfere with nasal inhalation, and clinical history of disordered eating that could interfere with food intake assessments. Recruitment methods included flyer placement, social media posts, and community outreach efforts, including presentations and focus groups. The most successful recruitment methods were placing flyers on the doors and in the mailboxes of churches, along with tabling at Walmart and other community locations populated heavily by African American persons.

### Study Design

#### Blinding and randomization

**Figure 1.**
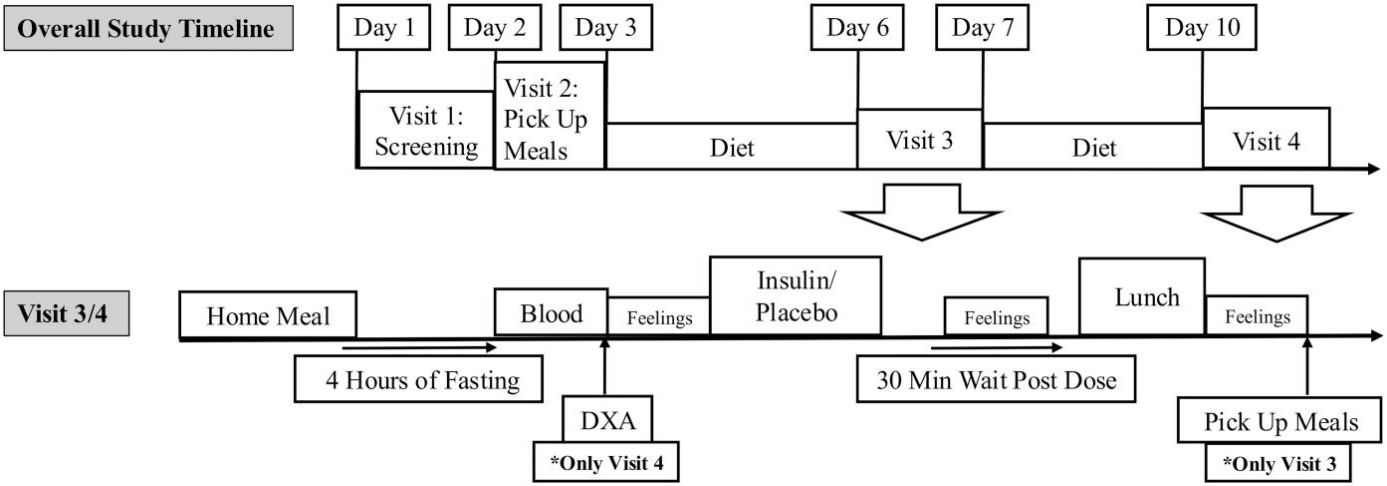
Schedule of study visits (top) and schedule of procedures during dosing visits (bottom). Participants completed 4 visits with a eucaloric lead-in diet preceding visits 3 and 4. Doses of insulin or placebo were administered at the third and fourth visits.

A double-blind, placebo-controlled, randomized crossover study design was used to compare a single acute dose of intranasal insulin to a saline placebo (Fig. 1). The order in which participants received insulin or placebo was randomized for visits 3 and 4. The insulin and saline vials were covered with a coded label made by the pharmacist to ensure that randomization was not broken by visual characteristics or existing labels on the vials. The pharmacist was the only member of the investigation staff who was not blinded to the drug, to allow for proper preparation of the drug delivery device. All other personnel directly involved in conducting the investigation (project manager, principal investigators, medical investigator, and participants) were blinded for the entire study.

The extent of the pharmacist's involvement in the study was preparation of insulin and placebo vials; that individual was not involved in collecting any study-related measurements from individuals. At the end of their participation in the study, participants were asked what order they believed they received the insulin and placebo doses, to assess the possibility that differences in sensation between insulin and placebo could have effectively unblinded them. Of the 40 participants, 12 correctly guessed the order in which they had received the nasal spray, suggesting that differences between placebo and insulin inhalation sensations were difficult to detect. Anecdotally, only a handful of participants reported a burning sensation or noteworthy taste on the day they received insulin.

#### Procedure

At the first clinical visit, all participants provided informed consent and completed a blood draw; metabolic hood (to estimate daily energy expenditure for the eucaloric lead-in diet); oral glucose tolerance test (OGTT); questionnaires about appetite, health history, demographics, family history of AD, and medications; and cognitive tests. At the second clinical visit, participants were given a 3-day eucaloric, nutritionally balanced lead-in diet (30% of calories from fat, 45% from carbohydrates, and 15% from protein), with the last of these meals consumed 4 hours prior to the third clinical visit. The third and fourth clinical visits included either a dose of intranasal insulin or placebo, followed by an ad libitum test lunch, with food-related feelings assessed via visual analog scales (VAS) before and after the dose as well as after the lunch. Another 3-day set of meals was provided during the third clinical visit, in preparation for the fourth clinical visit. Dual-energy x-ray absorptiometry (DXA) was completed during the fourth visit (body mass index [BMI] range, 24.6-49.6). Stratified randomization was used to randomize each participant to receiving insulin at the third clinical visit and placebo at the fourth clinical visit, vs the opposite ordering, while ensuring balance in the number of participants assigned to each ordering. The study protocol was approved and the study was overseen by the Institutional Review Board of Pennington Biomedical Research Center. The trial was registered at clinicaltrials.gov (NCT04739371).

### Intranasal Insulin Challenge

The intranasal ViaNase Device from Kurve Tech has been optimized for delivery of the spray to the nasal mucosa, facilitating direct delivery to the brain. The droplet size and unique vertical flow pattern of sprayed droplets are tailored to the specific characteristics of the sprayed drug to ensure that the dose is not lost to the lungs or stomach. Before the procedure began, the participant used a tissue to clear any mucous or blockage and remained seated for the duration of the procedure. The participant was asked to tilt their head back so the device could be inserted into the nostril to atomize 10 IUs (0.1 mL) of liquid (either Novolin-R insulin or saline placebo) into a fine mist. Each spray lasted a few seconds during which the participant was asked to sniff to aid delivery. This procedure was repeated twice in each nostril for a total of 40 IUs delivered per insulin dose. This dose of intranasal insulin was chosen based on data collected by one of the authors (K.L.G.) [41] in a clinical trial that examined the effects of intranasal insulin on cognition. Results showed that inhibitory control and sustained attention measures were enhanced in a dose dependent fashion, and the 40 IU dose used in the current study induced the greatest reductions in inhibitory control reaction time and sustained attention trail accuracy.

### Dual-energy x-ray absorptiometry

Dual-energy x-ray absorptiometry (DXA) scans were performed using the General Electric Lunar iDXA (General Electric; Milwaukee, WI) to determine body composition. Scans were analyzed using enCORE software version 13.60.033, resulting in measurements of fat mass and lean mass. Body fat percentage was calculated by taking the ratio of the DXA fat mass to the metabolic weight measured on a calibrated scale.

### Meals

#### Eucaloric lead-in diet

Calorie needs for the lead-in diet were calculated by multiplying the participant's resting metabolic rate by their activity factor, which estimates the participant's activity-related calorie expenditure. Activity factors ranged from 1.2 (sedentary) to 1.4 (active). Participants were instructed to eat all the food provided during the 3 days, and to not eat or drink any extra calories. The meals consisted of breakfast, lunch, and dinner and were the same at both time points. All meals were eaten within the participants' homes.

#### Test lunches

Participants were served ad libitum test lunches consisting of two 12″ sandwiches, regular potato chips (112.8 g), chocolate chip cookies (224.0 g), and water. Participants selected either ham, turkey, or roast beef sandwiches, and chose between fat-free mayonnaise or mustard in the amount of 10 g per sandwich. All sandwich types were similar in terms of energy and nutrient content. All participants were provided the same amounts of food and type of sandwich in each test meal. Participants were given an hour to eat and were instructed to eat until they were full. Provided food was covertly weighed prior to and after the test lunch.

### Visual Analog Scales

Visual analog scales (VAS) were used to measure participants' subjective ratings of hunger, fullness, desire to eat, and prospective food consumption as well as their desire to eat fatty, sweet, salty, and savory food types. The scales had a minimum value of 0 and a maximum value of 100. Questions included “How hungry do you feel at this moment?” “How full does your stomach feel at this moment?”, and “How thirsty do you feel at this moment?”.

### Data Analysis

Power analysis was conducted using G*Power (version 3.1.9.2.). An a priori power analysis on insulin-placebo differences with alpha set to .05 and power set at 0.80 was run to determine the sample size needed to detect smaller effect sizes (ie, Cohen's d = 0.5). A sample size of 40 participants was determined to be sufficiently powered assuming a 10% dropout rate. Linear mixed effect models were used to analyze differences in caloric intake and ingestive behaviors between intranasal insulin and placebo conditions. Sex and metabolic hood based respiratory quotient (RQ) were a priori covariates in each model, while period and sequence were also used for crossover effects based on the study design. Each outcome (total kcal, fat, carbohydrate, and protein intake at the test meal; proportion of calories consumed at the test meal that came from fat, carbohydrate, and protein; subjective ratings of hunger, fullness, desire to eat, and prospective food consumption; desire to eat fatty, sweet, salty, and savory food types) was analyzed in a separate model. Results are presented as least squares means with *t* tests used to test group differences, or beta coefficients. A set of sensitivity analyses repeated these same models, but using different sets of covariates in place of sex and RQ: sex and age; sex and estimated daily energy expenditure; sex and percent body fat; sex alone; and percent body fat and self-reported family history of AD. Because results were similar between the different models, the reported results are from the model that used sex and RQ as covariates. Output for all of the statistical models run has been provided in supplementary material [42]. A second set of sensitivity analyses was run excluding participants with a 2-hour fasting glucose value above 200 mg/dL on their OGTT. In all analyses, the significance level was set to .05.

## Results

### Participants

**Figure 2.**
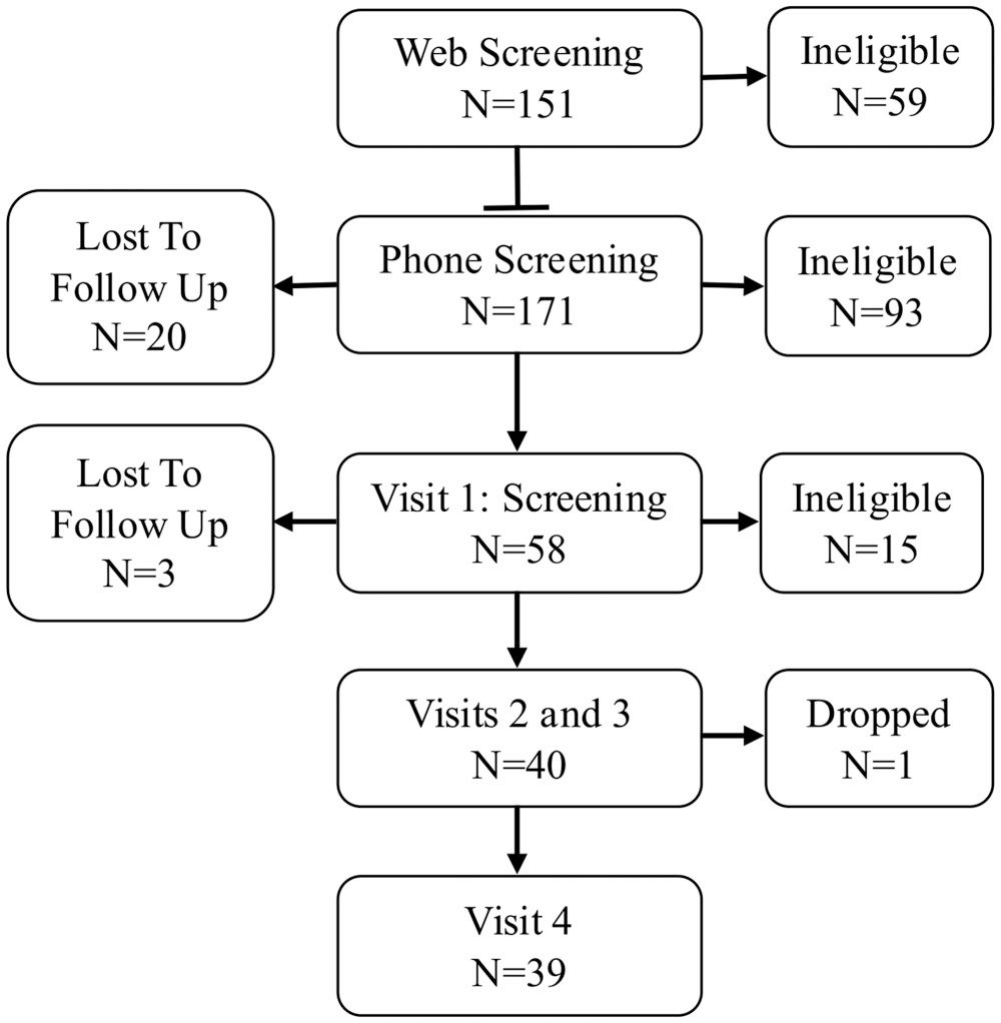
Flow diagram showing the number of individuals participating in each stage of study screening and visit completion.

A participant flow diagram for the study is shown in Fig. 2. A total of 39 middle-aged African American adults (71.8% female, aged 55.8 ± 5.6 years) completed the study (Table 1). Average body fat percentage was 44% ± 15.9%, 53.2% had undergraduate or graduate degrees, 59.6% were employed either full or part time, and 50.3% earned ≤ $50 000 per year.

**Table 1.** Summary of demographics, body composition, Matsuda Index, average OGTT 2-hour glucose measurements, average fasting glucose and insulin measurements, and resting metabolic rate data for all participants who completed at least 1 dosing visit

N	40
Age	55.8 ± 5.6 years
% Body fat	44.0 ± 15.9%
Average height	65.4 ± 3.61 inches
Average weight	217.9 ± 39 pounds
Average BMI	36.1 + 6.61 kg/m^2^
Education	53.2% had college or graduate degrees
Employment	59.6% employed full or part time
Sex	71.8% female
Family history of AD	18.6%
Income	55.3% at $50 k/year or below
Matsuda Index (N = 39)	4.03 ± 2.58
Average fasting glucose	90.28 ± 12.92 mg/dL
Average fasting insulin	10.38 ± 7.41 uU/dL
Average 2-hour OGTT glucose	167.50 mg/dL
DeltaTrac RMR (N = 32)	1516 kcal/day
Cosmed RMR (N = 5)	1593 kcal/day
Average days since last menstrual cycle (N = 4)	13 ± 11.52

Continuous values are reported as mean ± SD. Note that 32 participants had their RMR data measured from a DeltaTrac machine and 5 had their RMR data measured from a Cosmed machine; 2 participants were unable to complete the measurements, and in these individuals RMR was estimated formulaically using the Mifflin St. Jeor calculator based on age, height, and weight. Four female participants had regular menstrual cycles, and the average number of days since their last cycle is also included.

Abbreviations: AD, Alzheimer disease; BMI, body mass index; OGTT, oral glucose tolerance test; RMR, resting metabolic rate.

### Safety and Tolerability Data

No instances of clinically significant hypoglycemia were reported. An exit survey was conducted to assess discomfort with the nasal spray device. Three participants reported a burning sensation from the spray, and one individual reported that having something in their nose was unpleasant. However, no other complaints or complications were noted.

### Test Meal Results

There were no differences between insulin and placebo conditions in the total number of calories consumed (b = −29.76, SE = 34.09, DF = 28, t = −0.87, [95% CI, −99.59 to 40.07], *P* = .39), nor in the number of calories of protein (b = −4.87, SE = 5.06, DF = 28, t = −0.96, [95% CI, −15.23 to 5.49], *P* = .34), fat (b = −8.68, SE = 13.91, DF = 28, t = −0.62, [95% CI, −37.17 to 19.82], *P* = .54), or carbohydrates (b = −16.04, SE = 15.82, DF = 28, t = −1.01, [95% CI, −48.44 to 16.36], *P* = .32) consumed. Similarly, the proportions of calories that were consumed within protein categories (b = −0.30, SE = 0.35, DF = 28, t = −0.88, [95% CI, −1.02 to 0.41], *P* = .39), fat (b = 0.63, SE = 0.54, DF = 28, t = 1.16, [95% CI, −0.48 to 1.74], *P* = .26), and carbohydrate (b = −0.39, SE = −0.28, DF = 28, t = −1.38, [95% CI, −0.96 to 0.19], *P* = .18) did not differ between insulin and placebo conditions (Table 2). However, the total number of calories consumed did differ based on sex (F = 35.7, *P* < .0001), as did the total number of calories from protein (F = 47.09, *P* < .0001), fat (F = 23.09, < .0001), and carbohydrates (F = 36.93, < .0001).

**Table 2.** Left: total and macronutrient-specific intake during the test meals under placebo and insulin conditions, reported in kcals. Right: Proportion of total intake during the test meals that were accounted for by the 3 macronutrient categories, reported in percentages

Intake (kcals)				Proportion of total meal		
Macronutrient	Placebo	Insulin	*P* value	Placebo	Insulin	*P* value
Fat	280.63 (17.84)	271.95 (17.78)	.54	36.34%	36.97%	.26
Carbohydrates	361.31 (18.44)	345.26 (18.32)	.32	47.48%	47.09%	.18
Protein	119.45 (5.57)	114.59 (5.51)	.34	16.14%	15.84%	.39
Total	761.14 (39.20)	731.38 (38.99)	.39	—	—	—

Data are reported as mean (SE).

### Self-Reported Appetite Results

**Figure 3.**
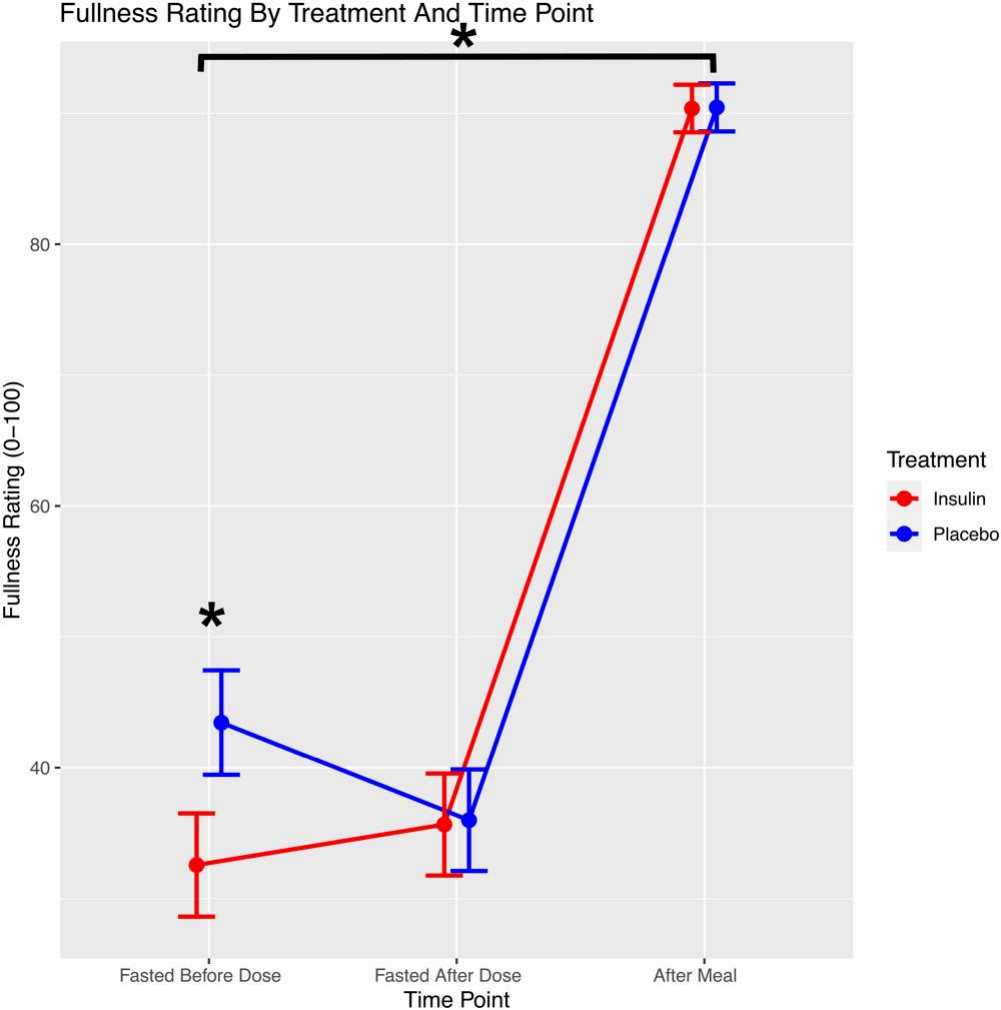
Increases in feelings of fullness between pre-dosing and post-meal time points were significantly greater in the insulin condition (red) compared to the placebo condition (blue). Fullness ratings were significantly higher among those about to inhale placebo, compared to those about to inhale insulin. *Significantly different between insulin and saline conditions, *P* < .05.

**Figure 4.**
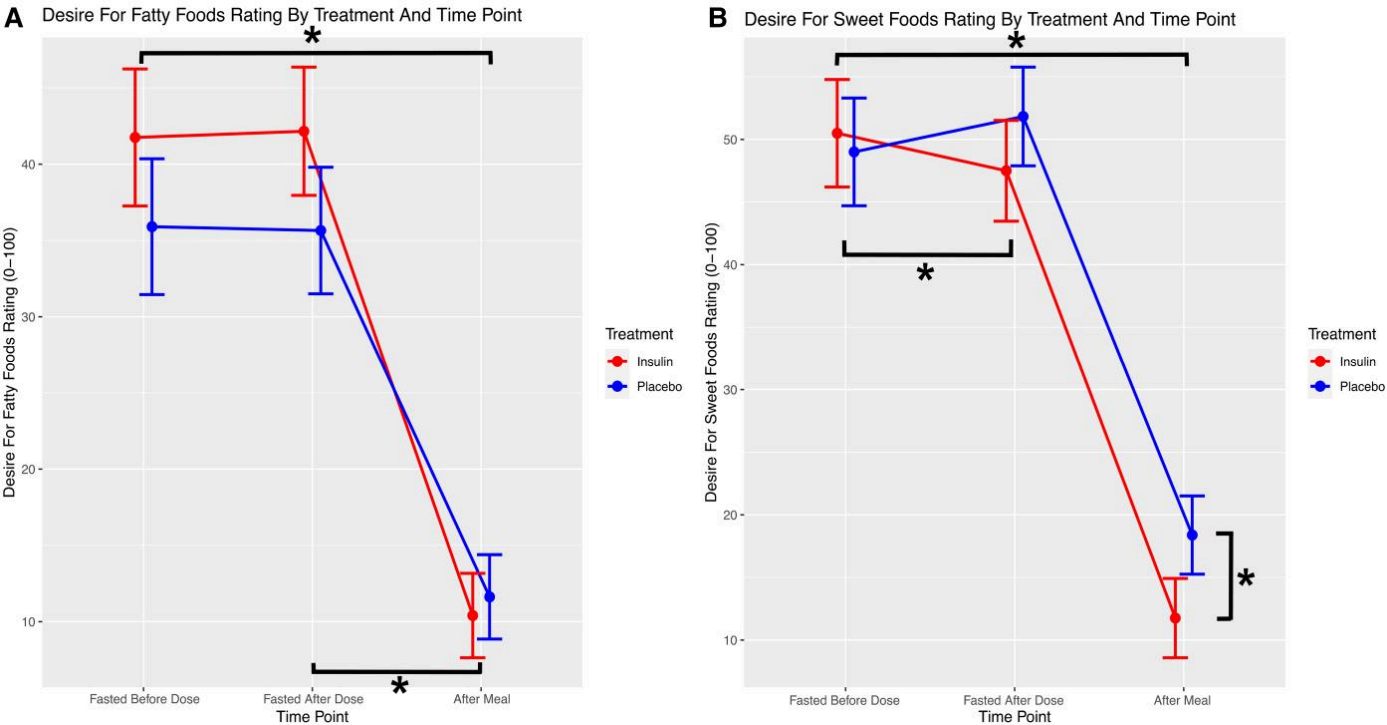
Self-reported desire to consume sweet foods reduced significantly more between pre- and post- insulin inhalation (red), than between pre- and post-placebo inhalation (blue). Similarly, the desire to eat sweet foods reduced more between pre-inhalation and post-meal time points when insulin was inhaled, compared to when placebo was inhaled. The post-meal desire to eat sweet foods was significantly higher among those who had inhaled placebo, compared to those who had inhaled insulin (Panel B). Additionally, the desire to consume fatty foods decreased significantly between pre-dosing and post-meal time points (Panel A). *Indicates significant differences at baseline and changes over time that are significant.

Mean scores on all VAS items before and after dosing, and after the test meal for insulin and placebo conditions respectively, are shown in Tables 3 and 4. Fullness ratings were significantly greater at the pre-dosing time point among those about to receive a dose of placebo, compared to those about to receive a dose of insulin (b = −10.89, SE = 4.38, DF = 28, t = −2.49, [95% CI, −19.85 to −1.92], *P* = .02, Fig. 3). The increase in fullness rating between pre-dosing and post-meal time points was significantly greater when receiving a dose of insulin compared to placebo (b = 10.81, SE = 4.56, DF = 28, t = 2.37, [95% CI, 1.47-20.14] *P* = .025, Table 5); the increase in this same rating between pre-dosing and post-dosing time points showed a trend toward being greater when receiving insulin compared to placebo (b = 10.56, SE = 5.71, DF = 28, t = 1.85, [95%CI −1.14 to 22.25], *P* = .08). In addition, there was a greater decrease in the desire to consume sweet foods between pre-dosing and post-dosing time points when receiving a dose of insulin compared to a dose of placebo (b = −6.45, SE = 3.06, DF = 28, t = −2.11, [95%CI −12.73 to −0.18], *P* = .04, Fig. 4). The desire to consume sweet foods additionally showed a trend toward greater decrease between pre-dosing and post-meal time points when receiving insulin compared to placebo (b = −8.44, SE = 4.55, DF = 28, t = −1.86, [95%CI −17.78 to 0.88], *P* = .07), and this same rating was significantly lower at the post-meal time point when receiving insulin compared to placebo (b = −4.85, SE = 2.33, DF = 28, t = −2.08, [95%CI −9.64 to −0.07], *P* = .04). The desire to consume fatty foods was significantly higher at pre-dosing (b = 10.28, SE = 4.20, DF = 28, t = 2.44, [95%CI 1.66-18.89], *P* = .021) and post-dosing (b = 11.42, SE = 4.39, DF = 28, t = 2.6, [95%CI 2.42-20.41], *P* = .01) time points when receiving insulin compared to placebo. Additionally, the desire to consume fatty foods decreased significantly between pre-dosing and post-meal time points (b = −11.92, SE = 4.40, DF = 28, t = −2.71, [95%CI −20.94 to −2.90], *P* = .01), and between post-dosing and post-meal time points (b = 13.06, SE = 4.71, DF = 28, t = −2.77, [95%CI −22.72 to −3.41], *P* = .01), when receiving insulin compared to placebo. This general pattern of findings was largely consistent across all sensitivity analyses (data not shown). Fullness ratings differed based on sex (F = 12.19, *P* = .002), as did perceived consumption (F = 7.73, *P* = .01), desire (F = 7.07, *P* = .01), and desire to consume sweet foods (F = 5.4, *P* = .03). Furthermore, these results were not significantly modified by excluding participants with clinically significant OGTT results (see Supplementary Tables S1-S4) [42].

**Table 3.** Self-reported ingestive behavior measurements before and immediately after dosing, and after the test meal, within the insulin dosing condition

VAS items	Fasted before dose	Fasted after dose	After meal	After dose vs before dose	After meal vs before dose
Hunger	50.04 (3.80)	54.58 (4.08)	7.78 (2.04)	4.54 (3.49)	−42.26 (4.32)
Fullness	32.57 (3.94)	35.66 (3.89)	90.36 (1.81)	3.09 (4.01)	57.79 (4.30)
Perceived Consumption	63.24 (2.51)	64.48 (2.97)	11.65 (2.30)	1.24 (2.58)	−51.58 (3.51)
Desire	59.87 (3.71)	61.07 (3.78)	10.83 (2.24)	1.29 (2.98)	−49.04 (4.18)
Fatty	43.47 (4.66)	43.26 (4.46)	7.92 (3.17)	−0.20 (2.35)	−35.54 (4.32)
Satisfied	39.30 (3.17)	38.27 (3.62)	85.09 (4.68)	−1.02 (3.24)	45.79 (5.27)
Sweet	50.11 (4.62)	46.48 (4.58)	11.28 (3.65)	−3.63 (2.06)	−38.82 (4.64)
Salty	45.40 (4.08)	43.67 (4.09)	12.02 (2.95)	−1.74 (1.92)	−33.39 (4.43)
Savory	63.70 (4.11)	60.46 (4.57)	18.04 (4.24)	−3.25 (2.89)	−44.36 (4.18)

All measurements were recorded on VAS scales with a minimum rating of 0 and a maximum rating of 100. Data are reported as mean (SE).

**Table 4.** Self-reported ingestive behavior measurements before and immediately after dosing, and after the test meal, within the saline dosing condition

VAS items	Fasted before dose	Fasted after dose	After meal	After dose vs before dose	After meal vs before dose
Hunger	44.12 (3.77)	51.73 (4.06)	7.26 (2.07)	7.60 (3.45)	−36.86 (4.29)
Fullness	43.46 (3.40)	35.99 (3.89)	90.44 (1.83)	−7.47 (4.02)	46.99 (4.38)
Perceived Consumption	63.45 (2.53)	64.70 (2.94)	9.66 (2.33)	1.25 (2.57)	−53.78 (3.56)
Desire	55.83 (3.72)	60.03 (3.76)	10.39 (2.26)	4.19 (2.99)	−45.44 (4.19)
Fatty	33.19 (4.66)	31.84 (4.44)	9.57 (3.18)	−1.35 (2.34)	−23.62 (4.33)
Satisfied	42.24 (3.18)	40.08 (3.59)	78.57 (4.73)	−2.16 (3.22)	36.33 (5.31)
Sweet	46.52 (4.62)	49.34 (4.54)	16.14 (3.65)	2.82 (2.04)	−30.37 (4.68)
Salty	43.41 (4.07)	41.50 (4.07)	11.33 (2.95)	−1.74 (1.92)	−33.39 (4.43)
Savory	57.98 (4.13)	60.03 (4.56)	18.04 (4.24)	2.05 (2.94)	−39.94 (4.20)

All measurements were recorded on VAS scales with a minimum rating of 0 and a maximum rating of 100. Data are reported as mean (SE).

**Table 5.** Between-arm differences in changes between pre-dose vs post-dose, as well as pre-dose vs post-meal

Between-arm differences		
VAS items	After dose vs before dose	After meal vs before dose
Hunger	−3.06 (4.23)	−5.39 (5.60)
Fullness	10.56 (5.71)	**10.81** (**4.56)**
Perceived Consumption	−0.01 (3.71)	−2.20 (3.89)
Desire	−2.99 (4.25)	−3.60 (5.47)
Fatty	1.14 (3.24)	**−11.92** (**4.40)**
Satisfied	1.14 (4.04)	9.46 (5.72)
Sweet	**−6.45** (**3.06)**	−8.45 (4.55)
Salty	0.17 (2.66)	−1.31 (5.19)
Savory	−5.30 (4.14)	−4.42 (4.48)

**Bold**: Significantly different between insulin and placebo conditions, *P* < .05. Data are reported as mean (SE).

## Discussion

The current study found that among obese, middle-aged African American adults, there were no significant differences in the amount or macronutrient content of food consumed after an acute dose of intranasal insulin, compared to an acute dose of an intranasal saline placebo. However, the acute dose of intranasal insulin was associated with greater decreases in self-reported preferences for fatty and sweet foods than the acute dose of placebo. Similarly, self-reported feelings of fullness were significantly greater after intranasal insulin ingestion, compared to placebo ingestion. This partially confirms our hypothesis that acute intranasal insulin administration would result in greater feelings of fullness, and less hunger. However, intranasal insulin did not result in the consumption of fewer calories. Overall, these findings suggest that the effects of a single intranasal insulin dose are small enough that food intake is not notably modified, yet feelings about food intake may change subtly, which could possibly culminate in gradual food intake changes over the course of repeated dosing.

The current study sample was obese on average, and a large literature has shown that significant weight loss confers numerous health benefits to young and middle-aged adults with obesity [43]. However, a significant amount of the weight that is lost (25%-33%) is muscle, not fat [44], and muscle loss late in the lifespan—ie, age-related sarcopenia—has emerged as a significant risk factor for adverse outcomes in older adults [45, 46]. As such, how to optimally balance the risks and benefits associated with weight loss among obese older adults is a topic of major research interest currently [44, 47]. With that said, if unintentional weight loss were a side effect of intranasal insulin prescribed to treat cognitive decline, it could be a significant concern. The relationship between adiposity and cognitive decline is complex [48, 49], with some studies suggesting that intentional weight loss could be associated with poorer cognitive function among older adults [50]. Among individuals diagnosed with AD, greater weight loss is correlated with faster disease progression, and loss of as little as 5% of body weight is associated with early mortality [28]. The acute effects we report on appetite among middle-aged African Americans are potentially concerning in this regard. As such, food intake changes over the course of repeated dosing should be investigated further as a possible side effect of chronic intranasal insulin exposure among middle-aged African Americans.

It is important to note that there was a discrepancy between feelings toward food consumption and actual food consumption. It is possible that this discrepancy is due to the acute nature of the intranasal insulin treatment in this study. For example, the dose of insulin may not have been high enough or administered for a long enough period of time to produce significant effects on food intake. Studies that have found significant effects of intranasal insulin on food intake have used doses of 160 IU [14, 15, 17]. Additionally, the samples in these studies are generally healthy weight young adults. Our sample differs in age and body composition, which may have influenced results. A study examining older adults found that daily dosing of 40 IU insulin for 24 weeks did not produce significant effects on food intake [27]. Another possible explanation for the lack of effect on food consumption in our study could be the gender composition of our sample. The sample was majority female, and women have been shown to be less sensitive to the effects of intranasal insulin on food intake [14, 17]. Notably, sex differences in food intake and certain VAS ratings were observed in our sample. Although both sets of results are important for understanding the effects of intranasal insulin, test meal results tell us more about what the participants actually ate in opposition to what they wanted to eat.

Also, the effects of chronic dosing that is more likely in an AD therapeutic setting are unclear. As mentioned above, the effects of intranasal insulin on food intake and self-reported feelings about food intake have been studied in both acute and chronic settings (see Schmid review [51]). But to our knowledge, no study to date has assessed both acute and chronic effects in the same individuals to determine whether acute responses are an accurate predictor of long-term ones, making speculation about the long-term implications of acute effects difficult. In fact, the lack of studies containing direct comparisons of acute to chronic responses seems to be a gap throughout much of the intranasal insulin literature, including studies of effects on cognition. There, effect sizes reported by chronic studies appear to be somewhat higher on average than those of acute studies (see, for example, Shemesh et al [52]), suggesting a possible accumulation of effects over the course of repeated dosing. However, future studies with chronic dosing within this same participant population are needed to understand whether any acute food intake related effects persist over the course of chronic dosing.

Because African Americans have been under-represented in intranasal insulin research to date, we focused on studying food intake side effects in this specific group, using community engagement efforts for recruitment. Our community engagement efforts used similar efforts to those employed at Alzheimer disease centers to establish African American research-ready cohorts, including face-to-face community presentations, focus groups to ascertain attitudes, beliefs, knowledge, and barriers to participation, and maintaining a continual community presence [53-55]. While these efforts have been promising, additional challenges remain; for example, similar to other efforts to increase engagement of African Americans in biomedical research, our cohort was limited by an overrepresentation of female participants [53] and highly educated individuals [55]. Additional work is needed to determine optimal approaches to enhance the representativeness of African American research cohorts with respect to both educational attainment and gender.

This study had noteworthy methodological strengths. The devices used to administer the doses were designed to eject liquid particles in a way that encouraged delivery to the brain rather than peripheral tissues, and the devices were calibrated to release the correct amount of insulin or saline at each dose. Individuals consumed a nutritionally balanced lead-in diet with daily caloric needs estimated by indirect calorimetry to reduce the effects of energy and nutrient imbalances as confounders of intranasal insulin effects. The use of community-based efforts to recruit a sample of individuals from a traditionally under-represented group is an additional strength. The study also had noteworthy limitations. The sample size was limited, thus reducing statistical power and precluding the recruitment of individuals from relevant comparator groups. As mentioned above, recruited African American individuals were not representative of the broader community in terms of income or education and included relatively few men. Finally, the study focused entirely on the effects of acute rather than chronic intranasal insulin exposure, although chronic exposure is commonly deployed in an AD-related therapeutic setting.

In conclusion, a single acute dose of intranasal insulin was associated with differences in feelings about food, but not test meal food intake, among middle-aged African Americans, compared to placebo.

## Data Availability

Some or all datasets generated during and/or analyzed during the current study are not publicly available but are available from the corresponding author on reasonable request.

## Clinical Trial Information

ClinicalTrials.gov registration no. NCT04739371

## Abbreviations

AD: Alzheimer disease
DXA: dual-energy x-ray absorptiometry
OGTT: oral glucose tolerance test
RQ: respiratory quotient
VAS: visual analog scale
